# Supplementary material for: Modeling of Growth and Organic Acid Kinetics and Evolution of the Protein Profile and Amino Acid Content during Lactiplantibacillus plantarum ITM21B Fermentation in Liquid Sourdough
Source: Foods. 2022 Dec 6;11(23):3942. doi: 10.3390/foods11233942 (PMC9741194; doi:10.3390/foods11233942)
Supplement: Supplementary file 1 [file foods-11-03942-s001.zip › foods-2010253-supplementary.pdf]

foods MDPI

**Table S1.** Growth rate datasets for the effects of temperature (**A**), pH (**B**) and water activity (**C**).

| | **T (°C)** | **$\mu_{max}$ ($h^{-1}$)** |
|---|---|---|
| **A** | 5.5 | 0.007 |
| | 11 | 0.087 |
| | 18 | 0.258 |
| | 18 | 0.241 |
| | 20 | 0.319 |
| | 20 | 0.288 |
| | 22 | 0.39 |
| | 22 | 0.392 |
| | 27 | 0.549 |
| | 27 | 0.553 |
| | 35 | 0.787 |
| | 35 | 0.769 |
| | 39 | 0.255 |
| | 39 | 0.235 |

| | **pH** | **$\mu_{max}$ ($h^{-1}$)** |
|---|---|---|
| **B** | 3.2 | 0.083 |
| | 3.2 | 0.089 |
| | 3.3 | 0.208 |
| | 3.3 | 0.217 |
| | 3.4 | 0.359 |
| | 3.4 | 0.362 |
| | 3.5 | 0.393 |
| | 3.7 | 0.489 |
| | 4.1 | 0.654 |
| | 4.6 | 0.662 |
| | 5.2 | 0.731 |
| | 5.8 | 0.736 |
| | 6.3 | 0.745 |
| | 6.8 | 0.706 |
| | 7.3 | 0.701 |
| | 7.6 | 0.672 |
| | 8 | 0.611 |
| | 8.7 | 0.585 |
| | 9.1 | 0.489 |

| | $a_w$ | $\mu_{max}$ ($h^{-1}$) |
|---|---|---|
| **C** | 0.996 | 0.786 |
| | 0.996 | 0.724 |
| | 0.992 | 0.674 |
| | 0.992 | 0.687 |
| | 0.989 | 0.638 |
| | 0.987 | 0.668 |
| | 0.98 | 0.479 |
| | 0.975 | 0.415 |
| | 0.975 | 0.402 |
| | 0.972 | 0.182 |
| | 0.97 | 0.189 |
| | 0.965 | 0.076 |
| | 0.965 | 0.06 |

**Table S2.** Growth rate datasets for the effects of undissociated lactic acid [*HA*] at pH 4.7 (**A**) and 5.1 (**B**)

| | pH | [*HA*] (mM) | $\mu_{max}$ ($h^{-1}$) |
|---|---|---|---|
| **A** | 4.7 | 0 | 0.415 |
| | 4.7 | 5 | 0.442 |
| | 4.7 | 5 | 0.452 |
| | 4.7 | 15 | 0.483 |
| | 4.7 | 15 | 0.464 |
| | 4.7 | 30 | 0.358 |
| | 4.7 | 30 | 0.393 |
| | 4.7 | 40 | 0.321 |
| | 4.7 | 40 | 0.312 |
| | 4.7 | 50 | 0.261 |
| | 4.7 | 50 | 0.292 |
| | 4.7 | 60 | 0.227 |
| | 4.7 | 60 | 0.197 |
| | 4.7 | 70 | 0.216 |
| | 4.7 | 70 | 0.185 |
| | 4.7 | 80 | 0.163 |
| | 4.7 | 80 | 0.157 |
| | 4.7 | 100 | 0.061 |
| | 4.7 | 100 | 0.104 |
| | **pH** | **[*HA*] (mM)** | **$\mu_{max}$ ($h^{-1}$)** |
| **B** | 5.1 | 0 | 0.507 |
| | 5.1 | 0 | 0.458 |
| | 5.1 | 5 | 0.488 |
| | 5.1 | 5 | 0.603 |
| | 5.1 | 15 | 0.543 |
| | 5.1 | 15 | 0.571 |
| | 5.1 | 30 | 0.487 |
| | 5.1 | 30 | 0.438 |
| | 5.1 | 40 | 0.413 |
| | 5.1 | 40 | 0.428 |
| | 5.1 | 50 | 0.374 |
| | 5.1 | 50 | 0.403 |
| | 5.1 | 60 | 0.323 |
| | 5.1 | 60 | 0.39 |
| | 5.1 | 70 | 0.315 |
| | 5.1 | 70 | 0.367 |
| | 5.1 | 80 | 0.338 |
| | 5.1 | 100 | 0.245 |
| | 5.1 | 100 | 0.294 |
